# Mammography screening in Tennessee: a tract-level spatial analysis of geographic and socioeconomic disparities

**DOI:** 10.1007/s10552-026-02190-9

**Published:** 2026-06-04

**Authors:** Sima Namin, Jonathan S. Wall, R. Eric Heidel, Ashton Brooks, Savannah Allen, Jennifer Ferris

**Affiliations:** 1 Office of Research Support, University of Tennessee Health Science Center, 1924 Alcoa Highway, Knoxville, TN 37920 USA; 2 Department of Surgery, College of Medicine-Knoxville, University of Tennessee Health Science Center, 1924 Alcoa Highway, Knoxville, Knoxville, TN 37920 USA; 3 Department of Medicine, College of Medicine-Knoxville, University of Tennessee Health Science Center, 1924 Alcoa Highway, Knoxville, TN 37920 USA

**Keywords:** Breast cancer screening, Health disparities, Appalachia, Rural health

## Abstract

**Purpose:**

Despite improvements in early detection, Tennessee ranks among the top ten states for breast cancer mortality among women. Mammography screening and early diagnosis are critical to reducing mortality, yet access and uptake vary widely across the State. We tested whether access to diagnostic services affected screening in the State.

**Methods:**

We merged CDC PLACES estimates of screening among women aged 50–74 with geocoded FDA-certified mammography facilities to derive drive-time categories. Tract covariates included education, poverty, insurance, race/ethnicity, primary care provider density, urban/rural status, and Appalachian designation. We mapped hot/cold spots using Getis-Ord Gi* and fit four nested beta-regression models: geography only, socioeconomic only, combined, and combined plus log-provider density.

**Results:**

Median screening prevalence was 74.8%. Hotspots clustered around Memphis, Nashville, and Knoxville; cold spots in Central Appalachian and western floodplain tracts. Geography explained ~ 23% of between-tract variation (pseudo-*R*^2^ = 0.238); socioeconomic covariates ~ 61% (0.607). In combined models, drive-time and Appalachian effects attenuated. Urban tracts screened 0.72 percentage points (pp) higher than rural, and each 1-pp rise in adults without a high-school diploma predicted − 0.13 pp.

**Conclusion:**

Urbanicity and education were the primary drivers of mammography screening disparities in Tennessee. To improve early diagnosis and outcomes, interventions should prioritize educational outreach, poverty reduction, insurance expansion, and improved geographic access, via mobile units or telehealth, targeting remote cold-spot tracts.

## Introduction

Breast cancer remains the second leading cause of cancer death among U.S. women, despite a 44% decline in mortality since 1989 largely due to widespread mammography screening programs [1]. Stage at diagnosis is a major prognostic factor and screening, which promotes earlier detection, significantly improves outcomes. The Surveillance, Epidemiology, and End Results (SEER) program reports a five-year relative survival of 100.0% for localized female breast cancer versus 32.6% for advanced stage [2]. The American Cancer Society (ACS), the American College of Radiology (ACR), and the National Comprehensive Cancer Network (NCCN) all recommend annual screening mammograms beginning at age 40 [3–5], yet national adherence hovers around 70% [6], leaving a substantial proportion of women at risk for advanced stage diagnosis.

While urban centers often achieve screening rates above the national average, rural and socioeconomically disadvantaged areas lag behind [7]. In Tennessee, breast cancer accounted for 14.7% of all cancer deaths and 28.8% of new cancer diagnoses between 2015 and 2019. Despite ranking 38th in incidence, the state ranked 6th in mortality and 12th in the mortality-to-incidence ratio, highlighting a disproportionately high mortality burden relative to its case burden placing the state sixth highest in female breast cancer mortality rate and 12th highest in the mortality‐to‐incidence ratio nationwide [8, 9]. These data underscore a critical need to understand and address gaps in mammography uptake, especially as Tennessee participates in the Centers for Disease Control and Prevention (CDC) and Centers for Medicare & Medicaid Services (CMS)‐funded screening initiatives (e.g., the National Breast and Cervical Cancer Early Detection Program) [10, 11].

The Appalachian region of Tennessee, characterized by mountainous geography and higher rates of poverty, faces pronounced access challenges. Geographic barriers such as long travel distances and transportation challenges [12] interact with socioeconomic factors [13] (low household income [14], insurance gaps [15], and provider shortages [16, 17]) to depress screening rates. County‐level analyses have documented lower early‐stage diagnosis and higher mortality in Appalachian versus non‐Appalachian counties [18].

Understanding where and why screening gaps persist is essential for guiding targeted interventions. Guided by Andersen’s Behavioral Model of Health Services Use [19], we conceptualize screening uptake as a function of predisposing (race/ethnicity), enabling (insurance, poverty, proximity to care, provider density), and subsequent need factors (local disease burden). The specific aims of this study were to

1. Map tract-level mammography screening prevalence across Tennessee and identify geographic disparities.
2. Examine associations between screening prevalence and tract-level sociodemographic, healthcare access, and geographic factors.
3. Identify underserved areas that may benefit from targeted interventions.

## Methods

### Study design

This study used a cross-sectional ecological design to examine tract-level mammography screening prevalence and its association with geographic access, socioeconomic characteristics, and provider availability across census tracts in Tennessee.

### Data sources

#### Mammography facilities

Locations of all the Food and Drug Administration (FDA)-certified mammography facilities for 2025 were obtained from the U.S. Food and Drug Administration’s publicly available dataset [20].

#### Screening prevalence

Crude estimates of mammography screening (women aged 50–74) were retrieved from CDC PLACES: Local Data for Better Health (2024), which derives tract-level “had a mammogram within the past two years” prevalence from the 2022 Behavioral Risk Factor Surveillance System (BRFSS)[21].

#### Population and socioeconomic data

Census tract centroids, population counts, and sociodemographic estimates were drawn from the U.S. Census Bureau’s Topologically Integrated Geographic Encoding and Referencing system (TIGER) shapefiles and the 2020 American Community Survey (ACS) 5-year estimates (2018–2022) [22]. CDC PLACES reports mammography screening prevalence for women aged 50–74 years. For access-related measures, we used women aged 40–74 to reflect current screening eligibility recommendations. For certain tract-level socioeconomic variables (e.g., poverty, insurance, race/ethnicity), exact ACS measures for women aged 50–74 were not consistently available, so women aged 45–74 were used as the closest approximation.

#### Provider data

Locations and specialty information for all practicing clinicians in Tennessee were obtained from the Centers for Medicare & Medicaid Services’ National Plan and Provider Enumeration System (NPPES) NPI file (July 2025 release) [23].

#### Appalachian counties

County boundaries designated as “Appalachian” were taken from the Appalachian Regional Commission’s (ARC) 2021 list [24].

#### Urban–Rural classification

We retrieved the tract‐level Rural–Urban Commuting Area (RUCA) data (2020) from the U.S. Department of Agriculture (USDA) Economic Research [25].

### Analysis

Mammogram facility addresses were geocoded and converted into a point shapefile using the tidygeocoder package [26] with the ArcGIS World Geocoding Service API. Population and socioeconomic data obtained from the American Community Survey (ACS) 5-Year Estimates included: Female Population 40–74 years—total number of women eligible for breast cancer screening; Female Population 45–74 years—denominator for calculating demographic and socioeconomic proportions; Race/Ethnicity—tract‐level percentages of Non-Hispanic White, Non-Hispanic Black, and Hispanic women aged 45–74; Poverty Rate—percentage of women aged 45–74 living below the federal poverty level; Uninsured Rate—percentage of women aged 45–74 without any health insurance coverage, and; Educational Attainment—percentage of women aged ≥ 25 without a high school diploma, with a bachelor’s or graduate degree.

For provider density, we filtered the full NPI file to five primary‐care taxonomy codes of interest: Family Medicine (207Q00000X), Internal Medicine (207R00000X), General Practice (208D00000X), Obstetrics & Gynecology (OB/GYN) (207V00000X), and Gynecologic Oncology (207VX0201X). The latter two were included because many women utilize their OB/GYN as a de facto primary care provider [27]. We then geocoded and spatially joined these points to respective tracts. We aggregated the number of providers per tract and calculated a Primary Care Provider (PCP) rate per 1,000 women age 40–74. For urban–rural classification, tracts were joined with RUCA data and tracts with Primary RUCA codes 1–3 were labeled Urban, and those with codes 4–10 were labeled Rural. Tracts were also flagged as Appalachian if they were within one of the ARC counties.

We then generated drive-time catchments for all 196 facilities using the ORS tool [28] in QGIS [29], producing isochrone polygons at 20, 30, 45, and 45 + minute thresholds. Each tract centroid was assigned to the nearest isochrone, and its female 40–74 population was attributed accordingly. Coverage was defined as the proportion of the tract’s eligible population within any service area. We computed coverage percentages for eligible women for each travel-time category.

To identify geographic clusters of high (“hot”) or low (“cold”) mammography screening prevalence, we conducted a local Getis-Ord Gi* hotspot analysis on tract‐level screening prevalence. We calculated a Gi*z‐score for each tract, which measures the degree to which each tract and its immediate neighbors exhibit clustering of high or low values relative to the statewide mean. Tracts with Gi* >  + 1.96 (*p* < 0.05) were classified as “hot spots,” those with Gi* < − 1.96 as “cold spots,” and all others as “not significant.” Tracts with missing screening data were coded “No data.”

For the statistical analysis, we first summarized tract‐level screening prevalence across four settlement types: Urban Appalachian, Urban Non-Appalachian, Rural Appalachian, and Rural Non-Appalachian, and across the three Tennessee “Grand Divisions” (East, Middle, West), by computing group means, standard deviations, medians, and boxplots. These visualizations created by ggplot2 [30] were provided to highlight disparities in screening prevalence prior to multivariable adjustment.

We used betareg [31] and emmeans [32] R packages to compare tract-level screening across settlement types. We fit an unadjusted beta-regression with a logit link and the four-level factor. We then used estimated marginal means on the response scale to obtain all six pairwise contrasts between groups, reported as percentage-point (pp) differences in mean predicted screening with 95% Wald confidence intervals and p values.

We then built four nested beta-regression models (logit link) to quantify the independent associations of access barriers, place, socioeconomic context, and provider supply with tract screening prevalence. Categorical factors were explicitly coded with reference levels (“≤ 20 min,” “Rural,” “Non- Appalachia”) and continuous predictors centered on percentages. Model performance was compared via McFadden pseudo-*R*^2^, Akaike Information Criterion (AIC), and Bayesian Information Criterion (BIC). This approach allowed us to isolate the contribution of geographic barriers, socioeconomic factors, and local provider supply-to-observed screening gaps.

## Results

Drive‐time access to mammography among women aged 40–74 varied markedly by settlement type. In urban non-Appalachian tracts, 86.3% lived within 20 min of a facility and only 1.1% exceeded 45 min. Urban Appalachian tracts showed a more dispersed profile (41.7% within 20 min; 40.0% at 21–30 min; 7.4% > 45 min). Rural non-Appalachian areas retained comparatively good access (64.8% within 20 min; 2.6% > 45 min), whereas rural Appalachian tracts faced the greatest barriers (38.7% within 20 min; 26.4% > 45 min). Collectively, these patterns demonstrate a pronounced urban–rural and Appalachian-non-Appalachian gradient with near-universal proximity in urban non-Appalachia versus substantial travel burdens in rural Appalachia.

County‐level analysis in Tennessee underscores these broad settlement‐type patterns with greater spatial detail. Counties encompassing major urban centers, Shelby (Memphis), Davidson (Nashville), Hamilton (Chattanooga), and Knox (Knoxville), fell in the highest access class (80–100% of women within a 20-min drive). In contrast, several rural Appalachian counties in eastern Tennessee (e.g., Hancock, Fentress, Scott,) exhibited 0% coverage within a 20-min drive.

Regarding settlement patterns, it should be noted that Black women aged 45–74 were clustered highly in just a few counties. Nearly half of the state’s population in this demographic (45.98%) lived in Shelby (53.4% Black) and Haywood Counties (52.8%). Every other county in TN exceeded 75% White in this age group, with a statewide average of ~ 90%. The greatest concentration of White populations occurred in the rural eastern and mid‐state counties.

**Fig. 1:**
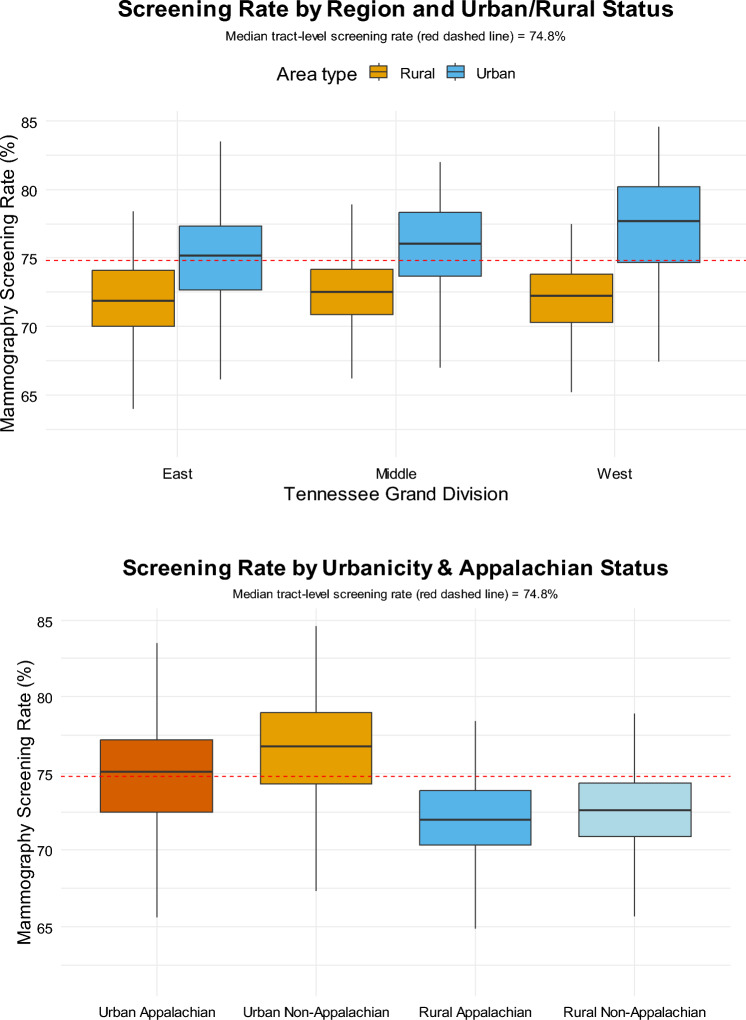
Mammography screening prevalence stratified by **A** Urban vs. Rural and **B** Appalachian vs. Non-Appalachian status

Across all three Grand Divisions (East, Middle, and West Tennessee), urban tracts consistently had higher mammography screening prevalence than rural tracts, and both sit above or very near the statewide median of 74.8%. Mammography screening prevalence differed systematically across the four categories defined by urbanicity and Appalachian status (Fig. 1). Urban non-Appalachian tracts exhibited the highest median screening (median ≈ 77%) whereas urban Appalachian had a median screening of (≈ 75%). Both rural non-Appalachian tracts had a median screening of ≈ 73%; and rural Appalachian tracts fell the most below the statewide median (median ≈ 74.8%).

Urban areas screened more than rural areas (Table 1). Urban non-Appalachian tracts averaged + 4.65 pp higher screening than rural Appalachian, and + 3.92 pp higher than rural non-Appalachian. Within urban tracts, non-Appalachia screened + 1.60 pp higher than Appalachia. All the differences were statistically significant, except that between rural Appalachian and rural non-Appalachian areas.

**Table 1:** Pairwise differences in mean screening (%)

Contrast (mean %)	Diff (pp)	z	95% CI	*p* value
Urban non-Appalachian vs Urban Appalachian	1.60	8.01	[1.07, 2.13]	< 0.001
Rural Appalachian vs Urban Appalachian	− 3.05	− 11.36	[− 3.76, − 2.35]	< 0.001
Rural Appalachian vs Urban non-Appalachian	− 4.65	− 18.66	[− 5.31, − 4.00]	< 0.001
Rural non-Appalachian vs Urban Appalachian	− 2.32	− 7.94	[− 3.09, − 1.55]	< 0.001
Rural non-Appalachian vs Urban non-Appalachian	− 3.92	− 14.27	[− 4.64, − 3.19]	< 0.001
Rural non-Appalachian vs Rural Appalachian	0.74	2.24	[− 0.13, 1.60]	0.025

Effects are percentage-point differences in mean predicted screening. CIs are 95% Wald

**Fig. 2:**
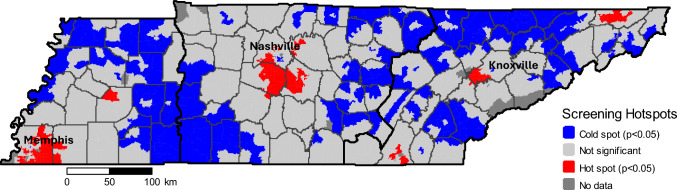
Regional variability in Mammography screening prevalence Across Tennessee Tracts

The Getis-Ord Gi* hotspot analysis revealed several distinct spatial patterns in Tennessee’s mammography screening prevalence (Fig. 2). Tracts where screening prevalence is significantly lower than their neighbors (*p* < 0.05; cold spots), were concentrated in rural areas, most notably in regions of Central Appalachia and the Eastern Highland Rim and in remote Western counties of Tennessee (for example, areas along the Mississippi River floodplain). Tracts with significantly higher screening prevalence than surrounding areas (hot spots), appeared primarily around urban centers and their suburbs (e.g., the Nashville, Knoxville, and Memphis metropolitan areas).

Next, we evaluated four nested linear beta-regression models (logit link) to further understand the factors that impact tract-level mammography screening prevalence in Tennessee (Table 2). In Model 1, we included only geographic predictors, i.e., drive time to the nearest facility (with ≤ 20 min as the reference), urban versus rural status, and an Appalachian indicator (yes/no). This model showed that screening declined monotonically with longer drive time: 21–30 min − 1.31 pp (p < 0.001), 31–45 min − 1.86 pp (*p* < 0.001), and > 45 min − 2.56 pp (*p* < 0.001) vs ≤ 20 min. Urban tracts screened + 2.94 pp more than rural (*p* < 0.001). Appalachian tracts screened − 1.32 pp compared with non-Appalachian (*p* < 0.001).

**Table 2:** Predictors of mammography screening prevalence in Tennessee

Variables	Model 1 geography & access	Model 2 socioeconomic	Model 3 model 1 &2	Model 4 model 3 + PCP rate
(Intercept)	73.57*** [73.16, 73.97]	74.78*** [74.67, 74.90]	74.22*** [73.93, 74.51]	74.24*** [73.96, 74.52]
Access 21–30 min vs ≤ 20 min	− 1.31*** [− 1.78, − 0.80]	–	− 0.30† [− 0.66, 0.04]	− 0.35† [− 0.69, 0.00]
Access 31–45 min vs ≤ 20 min	− 1.86*** [− 2.66, − 1.08]	–	− 0.11 [− 0.66, 0.42]	− 0.14 [− 0.68, 0.39]
Access > 45 min vs ≤ 20 min	− 2.56*** [− 3.60, − 1.56]	–	− 0.16 [− 0.86, 0.52]	− 0.18 [− 0.89, 0.51]
Urban vs Rural	2.94*** [2.55, 3.31]	–	0.72*** [0.43, 1.02]	0.70*** [0.42, 1.00]
Appalachia vs non-Appalachia	− 1.32*** [− 1.66, − 0.97]	–	0.25† [− 0.01, 0.51]	0.26† [− 0.01, 0.54]
% with Less Than High School Education (female aged ≥ 25)	–	− 0.13*** [− 0.15, − 0.11]	− 0.13*** [− 0.15, − 0.11]	− 0.13*** [− 0.15, − 0.11]
% with college degree (female aged ≥ 25)	–	0.08*** [0.07, 0.09]	0.08*** [0.07, 0.09]	0.08*** [0.07, 0.09]
% of women (45–74) Below Poverty	–	− 0.09*** [− 0.11, − 0.08]	− 0.09*** [− 0.10, − 0.08]	− 0.09*** [− 0.10, − 0.08]
% Of women (45–74) without Health Insurance	–	− 0.04*** [− 0.05, − 0.02]	− 0.04*** [− 0.06, − 0.02]	− 0.04*** [− 0.06, − 0.02]
% Of black (women 45–74) vs white	–	0.07*** [0.06, 0.07]	0.06*** [0.06, 0.07]	0.06*** [0.06, 0.07]
% of Hispanic (women 45–74) vs White	–	− 0.01 [− 0.03, 0.01]	− 0.02 [− 0.04, 0.01]	− 0.02 [− 0.04, 0.01]
Log rate of PCPs per 1 k women age 40–74				− 0.08 [− 0.18, 0.02]
AIC	− 6620	− 7727	− 7750	− 7750
BIC	− 6582	− 7684	− 7679	− 7674
pseudo-*R*^2^	0.238	0.607	0.615	0.615

^†^*p* < 0.1, *p < 0.05, **p < 0.01, ***p < 0.001

In Model 2, we focused solely on socioeconomic predictors including, educational attainment, poverty rate, insurance coverage, and racial composition. Here, we found that each 1 pp increase in the proportion of adult women without a high‐school diploma predicted a 0.13 pp decline in screening (*p* < 0.001), while each 1 pp increase in college‐educated women predicted a 0.08 pp rise (*p* < 0.001). Poverty and uninsurance also showed inverse associations: − 0.09 pp per + 1 pp poverty and − 0.04 pp per + 1 pp uninsured. A higher proportion of Black women was associated with slightly higher screening (+ 0.07 pp per + 1 pp Black), whereas the percentage of Hispanic women was not. Socioeconomic factors alone accounted for 60% of the between-tract variance in screening variation.

Model 3 combined geographic and socioeconomic variables. After adjustment, drive-time effects attenuated substantially, with only the 21–30 min category remaining marginally significant (− 0.30 pp, *p* < 0.10), while the 31–45 min and > 45 min categories were no longer significant. Urban tracts continued to have higher screening prevalence than rural tracts (+ 0.72 pp, *p* < 0.001). The Appalachian coefficient reversed direction (+ 0.25 pp, *p* < 0.10), suggesting that the disadvantage observed in Model 1 was largely explained by socioeconomic context. This combined model explained approximately 61% of the variance. In the final model, Model 4, adding log-PCP rate per 1,000 women (40–74) yielded a small, non-significant coefficient. Access coefficients remained non-significant; the urban advantage persisted (+ 0.70 pp, *p* < 0.001); Appalachia remained marginally positive (+ 0.26 pp, *p* < 0.10). Overall, the model fit was unchanged.

To assess nonlinearity in the association between primary care provider (PCP) density and screening, we replaced the single log-PCP term in Model 4 with a natural spline. The spline specification fit significantly better than the linear term (likelihood-ratio *χ*^2^(2) = 30.94, *p* < 0.001) and increased the pseudo-*R*^2^ from 0.615 to 0.623. All three spline-based functions were statistically significant (*p* = 0.002, < 0.001, and < 0.001, respectively). The adjusted response showed a slight increase at low PCP supply, then a plateau with a mild decline at higher supply. The magnitude was small: Predicted screening differed by ~ 0.5 percentage points between the 10th and 90th PCP percentiles. Consistently, the linear model’s average marginal effect was − 0.083 pp per + 1 unit in log (PCP rate + 1), indicating no meaningful overall linear association after adjustment.

## Discussion

Statewide tract-level analysis shows distinct geographic and socioeconomic patterns in mammography screening. As anticipated, cold spots cluster in remote rural tracts, especially Central Appalachia and the western floodplain, while hot spots concentrate around urban centers, notably Middle Tennessee near the NCI-designated Vanderbilt University Medical Center hub. Nashville and Memphis show stronger regional effects than Knoxville, likely reflecting larger catchments and denser screening capacity. Despite a statewide median screening prevalence near 75%, sizable pockets of under-screening persist, aligning with socioeconomic disadvantage and limited access to care [33].

Consistent with Andersen’s Behavioral Model [19], the nested regression models demonstrated that enabling factors, particularly socioeconomic context, drive the differences observed in screening prevalence. Model 2, which included only education, poverty, insurance, and racial composition, explained 60% of the variation, far outpacing the model that included only the geographic drive-time barrier to access (23%). We also observed a modest positive association between the tract‐level percentage of Black women and screening, a finding that at first glance might appear counterintuitive given well‐documented racial disparities in cancer care [34]. In Tennessee, however, over half of Black women aged 45–74 live in major metros (e.g., Memphis and Nashville) with higher facility access and outreach. This underscores the need to expand screening in rural, predominantly White Appalachian tracts to lift statewide rates.

In the fully adjusted Model 3, the initial negative association between Appalachian and reduced screening reversed, suggesting that the lower prevalence observed in Appalachia are largely attributable to underlying socioeconomic factors rather than geography per se. This suggests that addressing poverty, educational attainment, and insurance coverage can mitigate geographic disparities. Although geographic access effects attenuated substantially once socioeconomic factors were included, urbanicity remained a significant predictor of screening prevalence, though its magnitude was reduced. Urban tracts screened nearly 3 pp higher than rural in Model 1, but this advantage narrowed to < 1 pp after including socioeconomic context. Likewise, drive‐time effects lost statistical significance in the combined model. These results suggest that while reducing travel barriers e.g., via mobile mammography units or telehealth referrals, remain important, socioeconomic interventions may yield larger improvements in screening uptake.

Adding the local primary care provider density (Model 4) conferred almost no gains in model fit and exhibited only a small inverse association with screening (− 0.08 pp per log‐unit), consistent with a saturation effect whereby additional providers in already well‐served areas confer limited incremental benefit. This nonlinear relationship implies, predictably, that the greatest benefit would be afforded by efforts that prioritize PCP expansion in cold-spot tracts with very low provider density rather than scaling up across the state.

## Conclusion

The results demonstrate that socioeconomic factors, rather than geography alone, is the primary driver of the gaps in preventive care for breast cancer in Tennessee. While rural and Appalachian tracts initially appeared to lag behind their urban and non‐Appalachian counterparts, multivariable modeling revealed that these geographic disparities are largely explained by underlying differences in poverty, insurance coverage, and principally education.

Based on these analyses, efforts to increase mammography screening in Tennessee should prioritize social and economic interventions, such as investing in community health worker outreach, and delivering culturally tailored education, to improve screening. Geographic strategies, including mobile mammography units and telehealth navigation, remain valuable for the most remote cold‐spot tracts where travel burdens exceed 45 min; however, their marginal return may be modest compared to meaningful changes in socioeconomic factors, and particularly education. Ultimately, an integrated strategy that combines these factors is essential to ensure that all Tennessee women have access to life‐saving breast cancer screening.

## Limitations

Several caveats merit consideration. First, CDC PLACES provides modeled small-area estimates derived from Behavioral Risk Factor Surveillance System (BRFSS) survey data and census-based covariates rather than direct tract-level observations. As a result, screening prevalence estimates may be subject to model-based error or bias, particularly in sparsely populated areas. In addition, some independent socioeconomic variables in our analysis were derived from ACS data, which may introduce partial dependence due to shared underlying data sources. Second, the cross-sectional design captures a single time point and precludes causal inference between geographic, socioeconomic, or provider-supply factors and uptake. Third, provider density is derived from NPI taxonomy codes linked to tract centroids and does not capture program participation, part-time practice, or the extent to which OB/GYN and gynecologic-oncology clinicians function as de facto primary care providers. Fourth, assigning tract characteristics to a centroid assumes uniform within-tract population and access, potentially obscuring heterogeneity in demographics and travel times. Fifth, we use crude screening prevalence, which may affect comparisons across tracts with differing age structures. Sixth, ACS denominators and sociodemographic variables could not always be perfectly aligned with the BRFSS age band, necessitating adjacent groupings (women 45–74) and introducing possible misalignment. Despite these limitations, the multilayered spatial and statistical approach offers policy-relevant insight into the relative roles of place-based and socioeconomic determinants of mammography screening in Tennessee.

## Data Availability

This study used publicly available secondary datasets. Mammography facility data were obtained from the U.S. Food and Drug Administration Mammography Facility Database. Mammography screening estimates were obtained from CDC PLACES: Local Data for Better Health. Sociodemographic and geographic data were obtained from the U.S. Census Bureau American Community Survey 5-year estimates and TIGER/Line shapefiles. Provider data were obtained from the Centers for Medicare & Medicaid Services National Plan and Provider Enumeration System (NPPES) files. Rural–urban classifications were obtained from the U.S. Department of Agriculture Rural–Urban Commuting Area codes, and Appalachian county designations were obtained from the Appalachian Regional Commission. These datasets are publicly available through their respective websites.
